# Position Statement of the Union of European Phoniatricians (UEP): Fees and Phoniatricians’ Role in Multidisciplinary and Multiprofessional Dysphagia Management Team

**DOI:** 10.1007/s00455-022-10502-9

**Published:** 2022-08-16

**Authors:** Doris-Maria Denk-Linnert, Daniele Farneti, Tadeus Nawka, Antoinette am Zehnhoff-Dinnesen, Mieke Moerman, Patrick Zorowka, Mohamed Farahat, Antonio Schindler, Ahmed Geneid

**Affiliations:** 1 Division of Phoniatrics and Speech-Language Therapy, Department of Otorhinolaryngology, Head and Neck Surgery, Medical University of Vienna, University Hospital Vienna, Währinger Gürtel 18-20, 1090 Vienna, Austria; 2 Audiology and Phoniatrics Department - Romagna Health Service, Rimini Hospital, Rimini, Italy; 3 Department of Audiology and Phoniatrics, Charité-Universitätsmedizin, Berlin, Germany; 4 Department of Phoniatrics and Pedaudiology, University Hospital, Münster, Germany; 5 Private practice, Sint-Martens-Latem, Belgium; 6 Department of Hearing, Speech and Voice Disorders, Medical University, Innsbruck, Austria; 7 Department of Otolaryngology, Research Chair of Voice, Swallowing and Communication Disorders, College of Medicine, King Saud University, Riyadh, Saudi Arabia; 8 Department of Biomedical and Clinical Sciences, L. Sacco, Phoniatric Unit, University of Milan, Milan, Italy; 9 Department of Otorhinolaryngology and Phoniatrics-Head and Neck Surgery, University of Helsinki and Helsinki University Hospital, Helsinki, Finland

**Keywords:** Dysphagia management, Multidisciplinary and multiprofessional dysphagia team, FEES (flexible (fibreoptic) endoscopic evaluation of swallowing), Phoniatrics, Otolaryngology, Speech-language pathology

## Abstract

The need for multidisciplinary and multiprofessional management of dysphagia is constantly increasing and creating a major challenge for healthcare professionals and society, especially in terms of professional expertise and human resources. The distribution of tasks among the dysphagia team members, which includes phoniatricians, otolaryngologists, and speech-language therapists, is flexible and overlapping. For assessing dysphagia, the (fibreoptic) flexible endoscopic evaluation of swallowing (FEES), with or without videofluoroscopy, is a pivotal diagnostic tool. This position paper aims to illustrate the phoniatrician’s role in performing a FEES, which is an indispensable component of the diagnostic workup of patients suffering from oropharyngeal dysphagia. It is based on the current collaborative expert view of the Swallowing Committee of the Union of European Phoniatricians and a literature review. A FEES is one of the core competences of phoniatricians due to their endoscopic expertise and experience in the field of dysphagia and diseases of the upper aerodigestive tract. Therefore, the phoniatrician is an important member of the dysphagia team, for the medical diagnostics of the aerodigestive tract and dysphagia as well as for FEES. Phoniatric competence is especially important for head and neck cancer patients, infants, and complex cases.

## Introduction

In the last decades, dysphagia has become a great ethical and medical challenge for our society. Adequate management of the dysphagic patient requires a holistic, multidisciplinary, and multiprofessional team that includes phoniatricians, otorhinolaryngologists, speech-language pathologists (SLPs), gastroenterologists, neurologists, and intensive care physicians. Within the dysphagia team, the distribution of tasks is dynamic and overlapping. The purpose of this paper is to present the current view of the experts of the Swallowing Committee of the Union of European Phoniatricians on task sharing among the collaborating specialists involved in the field of dysphagia, in relation to flexible (fibreoptic) endoscopic evaluations of swallowing (FEES) and to illustrate the phoniatric responsibilities within the dysphagia team. The phoniatrician is a medical doctor specialized in the holistic management of swallowing, voice, communication, and paediatric hearing disorders and closely cooperates with SLPs in the field of behavioural treatment. However, internationally viewed, phoniatricians’ training, tasks, and responsibilities may vary, and worldwide phoniatrics is not always an independent specialty or subspecialty of ENT.

## Dysphagia and Its Significance

Dysphagia is defined as disturbance of the oral intake or transport of food from the oral cavity to the stomach. Depending on the impaired swallowing phases, a distinction is made between oropharyngeal (disturbed oral and pharyngeal phases) and oesophageal swallowing disorders, which may interfere with each other. Phoniatricians, otolaryngologists, and SLPs mostly focus on oropharyngeal dysphagia.

Although dysphagia is regarded as a symptom, it is more of a syndrome, with possible consequences such as compromised nutrition, general health, reduced life expectancy, impaired quality of life, psychosocial well-being, and limited participation. If aspiration is present, the affected patient is at risk of life-threatening aspiration pneumonia or chronic lung disease. Moreover, treatment and care have high costs [1–5].

The prevalence on dysphagia varies widely between 6 and 50% [6] and is on the rise. According to US statistics, 1 in 25 adults is annually affected by a swallowing disorder [7]. Dysphagia occurs at all ages but is most common among the elderly, with an estimated prevalence of 15–22% among those aged over 50, and of 60% or more in skilled nursing facilities [8, 9]. This is due to the age-related diminished functional reserve of the swallowing function, the increased occurrence of dysphagia-related morbidities, and dementia. For life expectancy, nutritional status, independent oral feeding, and prevention of aspiration-related pulmonary complications are highly relevant. Due to epidemiological development, the impact of dysphagia will continue to grow in the coming years. To date, geriatric oropharyngeal dysphagia has been underdiagnosed and underestimated. It is regarded as a geriatric syndrome and considered a geriatric giant which requires dedicated holistic intervention. [10–12].

Further reasons for the increased demand for dysphagia management are [7, 10, 13–20] as follows:

- advances in intensive care medicine, resulting in more patients surviving severe illnesses and undergoing long-term intubation and ventilation, possibly suffering from post-extubation- and ICU-related dysphagia
- advances in neonatology, which have increased the survival rate of very preterm infants and newborns with severe syndromic diseases who are at risk of compromised airways or dysphagia
- the enhanced use of organ-preserving therapy concepts in the treatment of head and neck cancer with the possible sequelae of aspiration-associated dysphagia.

## Dysphagia Diagnostics and Management

The possible aetiologies of oropharyngeal dysphagia include the following:

- Neurological diseases. Stroke and neurodegenerative diseases are the most common aetiologies among adults, whereas cerebral palsy is the most significant cause of dysphagia among children
- ICU-related dysphagia. The COVID-19 pandemic has created a new challenge for dysphagia management
- Structural diseases of the upper aerodigestive tract, e.g. head and neck malignancies and the sequelae after their treatment (surgery and (chemo-) radiotherapy), Zenker’s diverticulum, diffuse idiopathic skeletal hyperostosis, etc.
- Functional or psychogenic aetiology. This diagnosis is made by exclusion, and no abnormalities of the upper aerodigestive tract and /or swallowing physiology are detectable.

Dysphagia mostly demands a thorough morphological and functional diagnostic workup of the swallowing tract from the oral cavity to the stomach, because both oropharyngeal and oesophageal dysphagia may occur simultaneously. Due to the numerous aetiologies and consequences of dysphagia, the traditional boundaries between professional disciplines are crossed, and patient management often requires the contribution of many medical or therapeutic experts. A phoniatrician deals with oropharyngeal dysphagia together with an otorhinolaryngologist and SLP.

Since aspiration may occur silently, it must be safely proven or excluded only by direct visualization. The complementary dynamic instrumental diagnostic methods of FEES, which visualizes the pharyngeal phase of swallowing, and videofluoroscopy, which enables the assessment of all swallowing phases, have become the gold standard for dysphagia diagnostics [21].

FEES has become the most often used diagnostic tool for patients of all ages [22]. Due to the increased use of video endoscopes instead of fibrescopes, the term flexible endoscopic evaluation is more up to date for this process. The first description of a FEES in 1988, by Speech-Language Pathologist Susan Langmore and one of her co-workers, ENT Specialist Nels Olsen, reflects how FEES already had inherent interdisciplinarity in its early days [23].

The therapeutic armamentarium comprises causal treatment strategies such as surgery of a Zenker’s diverticulum or behavioural swallowing therapy by SLPs, including rehabilitative, compensatory techniques and manoeuvres, as well as dietary modifications and nutrition recommendations adapted to the individual swallowing disorder. Depending on the underlying aetiology and the individual dysphagia pathophysiology (dysphagia profile), an appropriate treatment regimen is individually tailored and based on evidence-based practice. The treatment aim is influenced by whether dysphagia has occurred as an acute illness or as a chronic or progressive disease, and it may be rehabilitative – to restore normal swallow functioning, or compensatory – with modifications of diet and patient behaviour [24].

## Phoniatrician’s Role in Multidisciplinary and Multiprofessional Dysphagia Team

### Team Approach

Due to the complexity and various etiologies of dysphagia, a multidisciplinary and multiprofessional team approach to holistic patient management is crucial*.* The dysphagia team encompasses several medical specialties, such as phoniatrics, otorhinolaryngology, neurology, radiology, gastroenterology, geriatrics, surgery, internal medicine, intensive care medicine, and therapeutic disciplines, such as speech-language pathology, dietology, and physical therapy. In recent decades, not only phoniatricians, otorhinolaryngologists, and SLPs have clinically and scientifically dealt with swallowing disorders; neurologists and geriatrics have also increasingly done so. This is reflected in the scientific literature and in education programmes. The introduction of a Master of Science in deglutology training programme at the University of Leuven and a UAB (Universitat Autònoma de Barcelona)-specific master’s degree in swallowing disorders at the University of Barcelona, which are both open to clinical professionals who have a degree in medicine or a health-related master's or bachelor’s degree (in speech- language pathology, physiotherapy, occupational therapy, or dentistry), make this interdisciplinarity evident.

The growing number of patients requires a broad-based competent dysphagia team for patient therapy and care. Screening methods, for which no gold standard yet exists, help identify patients who need an instrumental diagnostic swallowing evaluation. SLPs participate in the screening and clinical examination and are responsible for the behavioural (functional) treatment of oropharyngeal dysphagia.

### FEES: How, Who, and Distribution of Tasks

Dysphagic patients often first present to ENT specialists and phoniatricians. The phoniatrician does not only perform a diagnostic workup of the upper aerodigestive tract and the swallowing function, but, as a case manager, also coordinates the necessary interdisciplinary diagnostic and therapeutic procedure. In cases of suspected dysphagia/aspiration, a diagnostic instrumental investigation is essential, as is a thorough history examination and clinical swallowing evaluation. Performing a FEES does not only require endoscopic competencies, but it also requires extensive knowledge of dysphagia.

Overall, malignant diseases and structural pathologies must first be ruled out among dysphagic patients. Recently, it was observed that in patients after long-term intubation, ventilation, and tracheostomy for COVID-19 laryngeal sequelae and laryngotracheal stenoses did not occur rarely [25, 26]. This again underlines the need for a phoniatric /otolaryngological evaluation of the upper aerodigestive tract when performing a FEES.

According to the Langmore protocol, FEES should be carried out as standardized procedure [27]. Part 1 is the anatomical and physiological evaluation, Part 2 is the testing of food and liquids of different consistencies, and Part 3 is the testing of compensatory and adaptive treatment methods. Furthermore, for more comparable findings, the Secretion Severity Scale [28], the Penetration–Aspiration Scale [29], and the Yale Pharyngeal Residue Severity Rating Scale [30] should be used.

As a measurement of quality assurance, video recording helps enhance the reliability of FEES [31]. It has proved to be a reliable and well-tolerable procedure, with minor complications when performed by experienced otorhinolaryngologists, assisted by SLPs: the complication rate has been under 0.4%. Epistaxis, vasovagal syncope, or laryngospasm was observed in very few cases [32]. Moreover, Warnecke et al. described FEES as a well-tolerated and safe method for assessing swallowing function when performed by SLPs and neurologists in a stroke unit setting [33].

Task distribution within the dysphagia team must be viewed dynamically and is determined by historical country-specific developments of medical and therapeutic disciplines and legal regulations. Due to different national historical developments and practices, it is difficult to determine a generally accepted recommendation for task distribution [34]. In Anglo-American countries, where no phoniatric specialization exists, FEES is mainly performed by specialized SLPs, either independently or in conjunction with other members of the interprofessional team [24, 35]. SLPs perform FEES to analyse the swallowing function, not to establish a medical diagnosis. The American Speech Hearing Association (ASHA) does not require the presence of a physician for an SLP for a FEES [24]. In German-speaking and other European countries, including Italy and Poland, FEES is mainly performed by phoniatricians, for whom swallowing disorders are part of their core competence [36, 37]. Endoscopy including FEES is considered a non-delegable medical examination. Training curricula for phoniatricians and ENT specialists on dysphagia and FEES aim to guarantee good, standardized quality of education [38].

The rising patient numbers and the interest in dysphagia of disciplines other than phoniatrics has led other medical disciplines to start performing FEES (e.g. neurologists or geriatricians) and to establishing FEES curricula to guarantee the necessary skills and competencies [39]. The prerequisites required for qualification within the FEES curriculum of the European Society of Swallowing Disorders is two years of clinical practice in the care of neurological or geriatric patients for both doctors and healthcare professionals.

Who should perform FEES is determined by qualification-specific considerations rather than professional policy. This shows the necessary compromise between the increasing need for dysphagia management, available resources, and the best possible care. *The following aspects that underline phoniatric competence in performing FEES must be considered:*

- To perform a FEES examination, both clinical skills for handling an endoscope and knowledge on swallowing anatomy, (patho)physiology, and rehabilitation are required [35, 40]. Therefore, officially acknowledged training curricula should be established. Due to the expertise in endoscopy as well as in swallowing and voice disorders and the necessity to simultaneously examine the upper aerodigestive tract and the aetiology of dysphagia to make a medical diagnosis and to reveal individual swallowing pathophysiology, FEES should be performed by phoniatricians– at least when FEES is part of the initial examination. For phoniatricians, not only dysphagia, but also voice disorders are within the professional focus. Within the aerodigestive tract, its primary (vital) functions of breathing and swallowing and the secondary functions of phonation and articulation must be regarded as functional unit. Many diseases that cause dysphagia also cause voice disorders that must be treated. The phoniatrician recommends /performs the necessary conservative or surgical therapy. For example, the aim of phonosurgery in unilateral vocal fold paralysis is to enhance glottic closure through vocal fold augmentation or medialization. These procedures not only improve voice but also swallowing by reducing aspiration and enhancing clearance of aspirated material [41, 42]. The phoniatric expertise for voice and swallowing disorders makes the phoniatrician particularly competent in carrying out FEES and participating in dysphagia management, especially regarding the mutual influence of respiratory, vocal, and swallowing functions [43]. Furthermore, the phoniatrician has the professional overview to decide which type of swallowing therapy is indicated and when it should be chosen in challenging cases: behavioural treatment, drug therapy, or surgery (e.g. botulinum toxine application or myotomy to treat dysfunctions of the upper oesophageal sphincter, laryngeal suspension, laryngotracheal separation, tracheostomy, or total laryngectomy in cases of intractable aspiration).
- During the endoscopic diagnostic workup of the upper aerodigestive tract, malignant diseases or structural pathologies must be excluded. Patients are not etiologically ‘labelled’, and malignant or structural disease may also be present in neurological or geriatric patients. Diagnosis of the upper aerodigestive tract is a medical task for phoniatricians and ENT specialists [37, 44]. If the focus of dysphagia management is on the behavioural treatment of oropharyngeal dysphagia, FEES should ideally be carried out by a phoniatrician and/or an SLP**.** They then test compensatory (changes of posture, manoeuvres) and adaptive treatment techniques to provide essential information on useful therapy components for the treating SLP. In some institutions, FEES is performed by a phoniatrician in tandem with an SLP or by two examiners: one to perform nasopharyngolaryngoscopy and the other to perform the assessing /interpretation role. In clinical routine, a setting with two trained FEES examiners is not feasible everywhere due to staff shortages, and the second person often is an assistant. Video recording of FEES helps to meticulously analyse the examination and to improve the quality of findings [31].
- Moreover, the follow-up of head and neck cancer patients during behavioural swallowing treatment requires phoniatric expertise to, for example, detect tumour recurrence, make nutrition recommendations, or decide on decannulation if a tracheostomy tube is present. Moreover, tracheostoma care and tracheostomy tube management of the patient are an important part of dysphagia management and within the scope of the phoniatrician.
- Paediatric FEES: The management of dysphagia in paediatric patients is especially demanding, and in many cases, airway issues are also present. In this patient group, FEES is feasible, but procedural adaptations have been described for newborns. Due to the required special endoscopic expertise and experience, FEES should be carried out by phoniatricians. Early detection and management have a significant effect on development, health, and psychosocial aspects. So far, no standard protocol for paediatric FEES has been established [45–49].
- Patients with ICU-related dysphagia may suffer from laryngotracheal complications after long-term intubation and tracheostomy. In these cases, phoniatric expertise is valuable.
- In difficult local anatomical situations (e.g. very narrow nasal passage), FEES should be performed by the phoniatrician or otorhinolaryngologist.
  In view of the great need for dysphagia management and the limited number of phoniatricians, the performance of *FEES by non-phoniatric and non-otorhinolaryngological specialists* can be considered under certain circumstances:
- In cases of patients with neurogenic or geriatric dysphagia, neurologists/geriatricians may perform FEES only if they have the necessary skills and competencies. If the endoscopic findings are unclear, the patient must be referred to a phoniatrician or an otorhinolaryngologist.
- Ideally, the FEES training in the training programmes should be performed by phoniatricians / otorhinolaryngologists who have extensive endoscopic expertise
- How far SLPs can be involved in FEES or perform FEES in follow-up settings in institutions is dependent on the legal conditions in individual countries, their skills and competencies, the need/personnel resources, and the individual decision of the head of the department/hospital. A FEES training certificate does not overrule national regulations. Therefore, holders of either the FEES certificate or the FEES instructor certificate do not automatically acquire the right to carry out FEES in their home countries as this depends on national regulations [38]. SLPs work closely with phoniatricians and should be trained by them if needed for an approved training curriculum. The arguments in favour of follow-up FEES by SLPs are easier accessibility during treatment, direct control of therapeutic measures, and the use of FEES as a biofeedback tool in behavioural swallowing therapy [50, 51].

Even if FEES complications are rare, phoniatric /otorhinolaryngological support should be available, and in complex cases, the procedure should be conducted in a hospital setting. When SLPs perform FEES, they should know the contraindications and recognize in advance when the examination cannot be performed safely without medical assistance or office-based. They should identify any signs of adverse reactions and be prepared to react according to the specific protocols of their institution (52).

## Conclusion and Recommendations

The demand for multidisciplinary and multiprofessional management of dysphagia is increasing. The commitment of many disciplines involved leads to the present considerations and recommendations regarding FEES from the phoniatrician’s perspective (Table 1). Due to their expertise in endoscopy and diseases of the upper aerodigestive tract including swallowing and voice disorders, phoniatricians play a key role in the dysphagia team and, together with otorhinolaryngologists and SLPs, belong to its core members. Because of their special competence in swallowing disorders and behavioural treatment, as well as their clinical experience in diagnostics and treatment of organic and functional diseases of the upper aerodigestive tract, phoniatricians best meet the requirements for performing FEES as a prerequisite for a swallowing intervention.

**Table 1:** Considerations and recommendations regarding FEES from the phoniatrician’s perspective

•Phoniatricians meet the requirements to perform FEES in an optimal way due to their expertise in endoscopy and in diseases of the upper aerodigestive tract including dysphagia and voice disorders
•FEES should be carried out as standardized procedure. Video recording is recommended for quality assurance purposes •The great need for dysphagia management brings about the involvement of other medical disciplines in FEES especially in neurogenic or geriatric patients. Training curricula are necessary to guarantee a best possible standardized quality of training
•The involvement of SLPs in FEES depends on their skills, competencies, and legal regulations. A FEES training certificate does not overrule national regulations. The making of a diagnosis remains a medical task
•Patients with uncertain endoscopic findings or complex cases concerning the upper aerodigestive tract must be referred to a phoniatrician/ENT specialist

Due to increasing demand and growing interest, other medical specialists, such as neurologists or geriatricians, have started to perform FEES. These colleagues from partner disciplines are necessary to handle the large and increasing number of patients, although they have no comparable endoscopic expertise. The preconditions for medical professionals other than phoniatricians /ENT specialists to perform FEES are specialized training in accordance with approved training curricula for acquiring the necessary skills and competencies for treating dysphagia and performing FEES, and close cooperation with the phoniatrician /ENT specialist**.** In case of uncertain endoscopic findings during FEES, other professionals must refer the patient to a phoniatrician /ENT specialist. Phoniatricians have begun to offer interested medical disciplines FEES and dysphagia training courses.

Role distribution within the dysphagia management team is dynamic; it differs from country to country and is subject to developments and controversies. SLPs are dedicated partners of phoniatricians, and many of them have extensive expertise in dysphagia. They play an important role in screening, clinical examination, and behavioural dysphagia therapy. Depending on the history of the phoniatric discipline and legal regulations, SLPs perform FEES in many countries worldwide, especially in Anglo-American countries, where the phoniatric discipline does not exist. They have initiated efforts to also perform FEES in many parts of Europe. FEES Accreditation Courses in Europe are now also open to SLPs. An SLP’s involvement in FEES is useful if it is in close cooperation with phoniatricians and in the case of follow-up examinations for therapeutic purposes other than diagnostics. How far specially trained SLPs are involved in FEES depends on legal regulations and is decided on by the head of the individual medical institution for which they work on an individual basis. It also depends on medical necessity, the personnel situation, and their expertise.

However, for patients with head and neck cancer or tracheal cannula, paediatric patients, and patients with ICU-related dysphagia, it is recommended that FEES is performed by a trained phoniatrician or laryngologist.

In the future, joint research will be needed to establish standards for dysphagia diagnostics and treatment.
